# A novel natural variation in the promoter of *GmCHX1* regulates conditional gene expression to improve salt tolerance in soybean

**DOI:** 10.1093/jxb/erad404

**Published:** 2023-10-21

**Authors:** Yang Li, Heng Ye, Tri D Vuong, Lijuan Zhou, Tuyen D Do, Sushil Satish Chhapekar, Wenqian Zhao, Bin Li, Ting Jin, Jinbao Gu, Cong Li, Yanhang Chen, Yan Li, Zhen-Yu Wang, Henry T Nguyen

**Affiliations:** Institute of Nanfan & Seed Industry, Guangdong Academy of Science, Guangzhou, 510316, China; Division of Plant Science and Technology, University of Missouri, Columbia, MO 65211, USA; National Key Laboratory of Crop Genetics and Germplasm Enhancement, National Center for Soybean Improvement, Key Laboratory for Biology and Genetic Improvement of Soybean (General, Ministry of Agriculture), Jiangsu Collaborative Innovation Center for Modern Crop Production, Nanjing Agricultural University, Nanjing, 210095, China; Division of Plant Science and Technology, University of Missouri, Columbia, MO 65211, USA; Division of Plant Science and Technology, University of Missouri, Columbia, MO 65211, USA; Division of Plant Science and Technology, University of Missouri, Columbia, MO 65211, USA; Division of Plant Science and Technology, University of Missouri, Columbia, MO 65211, USA; Division of Plant Science and Technology, University of Missouri, Columbia, MO 65211, USA; National Key Laboratory of Crop Genetics and Germplasm Enhancement, National Center for Soybean Improvement, Key Laboratory for Biology and Genetic Improvement of Soybean (General, Ministry of Agriculture), Jiangsu Collaborative Innovation Center for Modern Crop Production, Nanjing Agricultural University, Nanjing, 210095, China; National Key Laboratory of Crop Genetics and Germplasm Enhancement, National Center for Soybean Improvement, Key Laboratory for Biology and Genetic Improvement of Soybean (General, Ministry of Agriculture), Jiangsu Collaborative Innovation Center for Modern Crop Production, Nanjing Agricultural University, Nanjing, 210095, China; National Key Laboratory of Crop Genetics and Germplasm Enhancement, National Center for Soybean Improvement, Key Laboratory for Biology and Genetic Improvement of Soybean (General, Ministry of Agriculture), Jiangsu Collaborative Innovation Center for Modern Crop Production, Nanjing Agricultural University, Nanjing, 210095, China; Institute of Nanfan & Seed Industry, Guangdong Academy of Science, Guangzhou, 510316, China; Institute of Nanfan & Seed Industry, Guangdong Academy of Science, Guangzhou, 510316, China; Institute of Nanfan & Seed Industry, Guangdong Academy of Science, Guangzhou, 510316, China; National Key Laboratory of Crop Genetics and Germplasm Enhancement, National Center for Soybean Improvement, Key Laboratory for Biology and Genetic Improvement of Soybean (General, Ministry of Agriculture), Jiangsu Collaborative Innovation Center for Modern Crop Production, Nanjing Agricultural University, Nanjing, 210095, China; Institute of Nanfan & Seed Industry, Guangdong Academy of Science, Guangzhou, 510316, China; Division of Plant Science and Technology, University of Missouri, Columbia, MO 65211, USA; Oklahoma State University, USA

**Keywords:** *cis*-element, *GmCHX1*, QTL mapping, salt tolerance, soybean

## Abstract

Identification and characterization of soybean germplasm and gene(s)/allele(s) for salt tolerance is an effective way to develop improved varieties for saline soils. Previous studies identified *GmCHX1* (*Glyma03g32900*) as a major salt tolerance gene in soybean, and two main functional variations were found in the promoter region (148/150 bp insertion) and the third exon with a retrotransposon insertion (3.78 kb). In the current study, we identified four salt-tolerant soybean lines, including PI 483460B (*Glycine soja*), carrying the previously identified salt-sensitive variations at *GmCHX1*, suggesting new gene(s) or new functional allele(s) of *GmCHX1* in these soybean lines. Subsequently, we conducted quantitative trait locus (QTL) mapping in a recombinant-inbred line population (Williams 82 (salt-sensitive) × PI 483460B) to identify the new salt tolerance loci/alleles. A new locus, *qSalt_Gm18*, was mapped on chromosome 18 associated with leaf scorch score. Another major QTL, *qSalt_Gm03*, was identified to be associated with chlorophyll content ratio and leaf scorch score in the same chromosomal region of *GmCHX1* on chromosome 3. Novel variations in a STRE (stress response element) *cis*-element in the promoter region of *GmCHX1* were found to regulate the salt-inducible expression of the gene in these four newly identified salt-tolerant lines including PI 483460B. This new allele of *GmCHX1* with salt-inducible expression pattern provides an energy cost efficient (conditional gene expression) strategy to protect soybean yield in saline soils without yield penalty under non-stress conditions. Our results suggest that there might be no other major salt tolerance locus similar to *GmCHX1* in soybean germplasm, and further improvement of salt tolerance in soybean may rely on gene-editing techniques instead of looking for natural variations.

## Teaser

A novel variation in *GmCHX1* promoter regulates conditional gene expression to improve salt tolerance for an energy-cost efficient strategy to protect soybean yield in saline soils.

## Introduction

Soil salinization that limits crop production is a global issue in agriculture (Munns and Tester, 2008; Gong *et al*., 2020; Li *et al*., 2023), and more than 45 million hectares accounting for at least 20% of total irrigated land has been affected by salt stress (Lauchli *et al.*, 2008; Xiao and Zhou, 2022). This problem is being exacerbated and accelerated by adoption of irrigation (Carillo *et al.*, 2011; Ondrasek *et al.*, 2011; Sahab *et al*., 2021), and salt-affected farmland is predicted to double by the year 2050. In salinized conditions, plants suffer osmotic stress first, and then ionic stress associated with sodium (Na^+^) and chloride (Cl^−^) accumulation (Deinlein *et al.*, 2014; Do *et al.*, 2016, 2018; Wei *et al.*, 2016; Wang *et al*., 2020; Yin *et al*., 2023). As a major legume crop, soybean (*Glycine max* (L.) Merr.) is widely grown and consumed for its high oil and protein contents (Singh, 2010; Duan *et al.*, 2022). Under salt stress, the accumulation of Na^+^ and Cl^−^ in soybean stems and leaves eventually leads to leaf chlorosis and necrosis, decrease of plant biomass, and yield reduction (Essa, 2002; Lee *et al.*, 2008; Lu *et al*., 2021). Soybean yield could be reduced by 20–50% due to salt stress (Lee *et al.*, 2008; Patil *et al.*, 2016). To overcome the negative impacts of salt stress in soybean, it is essential to discover genetic resources and to understand the mechanism of salt tolerance (Leung *et al*., 2023).

A major salt tolerance gene, *GmCHX1/GmSALT3/GmNcl*, was mapped and cloned from both wild and cultivated soybeans (Guan *et al.*, 2014; Qi *et al.*, 2014; Do *et al.*, 2016). *GmCHX1* encodes Cation/Proton Antiporter 2 (CPA2), a transporter with tissue-specific expression in phloem and xylem cells that exports both Na^+^ and Cl^–^ ions from xylem and reduces the accumulation of both ions in the shoot in saline conditions, but with extra energy cost (Guan *et al.*, 2014; Qi *et al.*, 2014; Liu *et al.*, 2016; Cao *et al.*, 2018). Two major functional variations were identified in *GmCHX1*: a 3.78-kb retrotransposon insertion in the third exon of *GmCHX1* that led to non-functional *GmCHX1* (Guan *et al.*, 2014; Qi *et al.*, 2014; Qu *et al.*, 2022) and a 148/150-bp insertion in the *GmCHX1* promoter, which may affect the transcriptional regulation of *GmCHX1* through disrupting the effect of *cis*-element located upstream of the insertion site, that led to lower *GmCHX1* expression (Guan *et al.*, 2014; Nakano *et al.*, 2020). A haplotype analysis of *GmCHX1/GmSALT3* in 172 soybean lines revealed almost all salt-tolerant lines have no retrotransposon insertion in the third exon and no 148/150-bp insertion in the promoter, in contrast to most sensitive lines (Guan *et al.*, 2014). The expression of *GmCHX1* in the tolerant lines is consistently higher than that in the sensitive lines under both control and salt stress conditions, which could result in unnecessary energy cost with yield penalty under non-stress conditions (Guan *et al.*, 2014; Qi *et al.*, 2014). Expression and silence of the stress resilience genes under stress and non-stress conditions, respectively, is desirable to avoid unnecessary energy cost (Lin *et al.*, 2003; Zhu *et al.*, 2010). Stress-inducible promoters could be one strategy to precisely control the expression of stress resilience genes in different environments.

Other than the major locus on Chr. 03 harboring *GmCHX1* gene (Lee *et al.*, 2004; Hamwieh and Xu, 2008; Hamwieh *et al.*, 2011; Ha *et al.*, 2013; Do *et al.*, 2018), a few loci associated with soybean salt tolerance were identified on Chrs 06, 07, 08, 10, 13, 15, 18, and 19 that explained the smaller phenotypic variation of each locus (Lee *et al.*, 2004; Chen *et al.*, 2008; Do *et al.*, 2018, 2019; Cho *et al*., 2021; Guo *et al*., 2021). Therefore, the question of whether there is any other major salt tolerance locus in the soybean germplasm needs to be answered. The objectives of this study were to identify novel genetic resources (loci or alleles) for salt tolerance in soybean and to investigate a more energy cost efficient strategy to manage salt stress in soybean production. A new quantitative trait locus (QTL) for salt tolerance was identified on Chr. 18 in the recombinant inbred line (RIL) population of Williams 82 × PI 483460B (wild soybean). A novel *GmCHX1* allele with salt stress-inducible promoter was identified in the same population, which provides a new genetic resource to develop an energy cost efficient (conditional gene expression) strategy to protect soybean yield in saline soils and non-stress conditions.

## Materials and methods

### Plant materials and growth conditions

Two soybean salt-tolerant lines, Lee and Fiskeby III, and three salt-sensitive lines, Jackson, Hutcheson, and Williams 82 (Luo *et al.*, 2005; Do *et al.*, 2018; Valliyodan *et al.*, 2021), were used as checks in this study. Also, seven additional salt-tolerant lines were selected from the Nguyen Lab’s core soybean germplasm of 305 accessions as previously reported (Do *et al.*, 2019). They included four wild soybean lines and three cultivated soybean lines.

The 182 F_7:8_ RILs derived from a cross of Williams 82 × PI 483460B as previously described (Patil *et al.*, 2018) were used to analyse the salt tolerance QTLs in this study. The parental lines, Williams 82 and PI 483460B, are classified as maturity group III. The hybrids were made at the Fisher Delta Research Center, University of Missouri, Portageville, MO, USA and the single seed descent method was used to develop the mapping population. Plants were grown under a cycle of 16 h (26 °C) day–8 h (22 °C) night in a greenhouse at the University of Missouri, Columbia, MO, USA.

### Evaluations for salt tolerance traits

Soybean plants were tested for salt tolerance using an established greenhouse method described by Lee *et al.* (2008) with minor modifications. Briefly, seven seedlings of each line at the vegetative growth stage V2, grown in Cone-tainers, were subjected with 100 mM NaCl solution. For the RIL population, the assays were conducted with two biological replicates and each replicate included seven seedlings per RIL. The other tested lines were evaluated with three biological replicates and each replicate had seven seedlings per line. When the two salt-sensitive checks, Jackson and Hutcheson, showed severe leaf scorch (approximately 2 or 3 weeks after the NaCl treatment), individual soybean plants were rated for leaf scorch score (LSS) using a 1–5 scale as previously described (Do *et al.*, 2018), where 1 is no apparent chlorosis, 2 is slight chlorosis (25% of the leaves showed chlorosis), 3 is moderate chlorosis (50% of the leaves showed chlorosis and some necrosis), 4 is severe chlorosis (75% of the leaves showed chlorosis and severe necrosis), and 5 is dead (leaves showed severe necrosis and were withered).

Leaf chlorophyll content (CC) was quantified on individual plants using a chlorophyll meter (Chlorophyll meter SPAD-502, Konica Minolta). The leaf chlorophyll contents were measured before and after NaCl treatment (CC_CK and CC_Salt). Chlorophyll content ratio (CCR) was calculated as:


$$\begin{array}{l} CCR=CC\;\_\;Salt/CC\;\_\;CK. \end{array}$$

### DNA, RNA isolation and quantitative real-time PCR

Genomic DNA was isolated from soybean leaves using the CTAB method (Murray and Thompson, 1980). Total RNA was isolated from root samples using the RNeasy Mini Kit (Qiagen, Hilden, Germany). Quantitative real-time PCR (qRT-PCR) was performed in a total volume of 15 μl with 2 μl diluted cDNA, 7.5 μl 2×SYBR, and 0.3 μM of gene-specific primers. The specific primers of *GmCHX1* were designed and the housekeeping gene *GmUKN1* (*Glyma.12G020500*) was used as an internal control (Guan *et al.*, 2014). The sequences of primer pairs are listed in Supplementary Table S1. The qRT-PCR reactions were run under the following procedure: initial denaturation at 95 °C for 5 min, 40 cycles of denaturation at 95 °C for 10 s, annealing at 60 °C for 20 s, and extension at 72 °C for 20 s. The Roche 480 Realtime detection system (Roche Diagnostics, Switzerland) was used for the segment amplification and data analysis. The and methods were used to calculate the relative gene expression in transcript levels (Livak and Schmittgen, 2001).

### Genotype analysis for *GmCHX1* gene

Kompetitive allele specific PCR (KASP) assays were developed for three gene-based markers (GBMs) to genotype the tested soybean lines for *GmCHX1*. The markers Salt-20, Salt14056, and Salt11655 were designed for single nucleotide polymorphisms (SNPs) in the promotor, the third intron, and the fifth exon of *GmCHX1*, respectively, as previously described (Patil *et al.*, 2016). The insertion–deletion (InDel) of 148/150 bp in the promoter of *GmCHX1* was genotyped by PCR and agar gel electrophoresis using specific primers (Supplementary Table S1). For the 3.78 kb InDel in the third exon of *GmCHX1* (Guan *et al.*, 2014), cDNA was used as the template and specific primer pairs were used for PCR (Supplementary Table S1).

### Linkage map construction and quantitative trait locus analysis

A bin map of the RIL population with 182 RILs and 4070 bins was available as previously reported by Patil *et al.* (2018). The program MapQTL 5.0 was initially used to detect the putative QTL by the interval mapping method (Van Ooijen, 2004). Composite interval mapping was then performed using the multi-QTL method (Van Ooijen, 2004). A significant threshold of logarithm of the odds (LOD) score was calculated for each trait by 1000 permutations to determine a QTL at the linkage group and genome-wide significance level of *P*=0.05 (Doerge and Churchill, 1996). Nomenclature of the identified QTL followed the SoyBase guidelines, where *qSalt_Gm* stands for *qtl_Salt Tolerance_Glycine max_chromosome number*. The epistatic interaction between salt tolerance QTL was analysed by the QTLNetwork 2.1 program (Yang *et al.*, 2008).

### Dual-luciferase reporter assay for promoter activity

Promoters of *GmCHX1* from different soybean lines were cloned and sequenced. The sequence variation was detected by BLAST between different soybean lines, and *cis*-elements were analysed by PlantCARE (http://bioinformatics.psb.ugent.be/webtools/plantcare/html/). The sequence of the *GmCHX1* promoter was cloned into the reporter vector of pGreenII 0800-Luc by One Step Cloning Kit (Vazyme, China) using *Kpn*I and *Xho*I restriction sites. The recombinant plasmids were transformed into *Agrobacterium tumefaciens* GV3101. After culture, the GV3101 inoculums containing the reporter gene were injected into tobacco leaves. The infiltrated plants were grown under control or 100 mM NaCl treatment for 48 h. Subsequently, the luciferase activities were measured with a dual-luciferase reporter assay kit (Vazyme). The primer pairs used in this assay are listed in Supplementary Table S1.

### Promoter-*β-glucuronidase* assay in soybean hairy root transformation

The *cis*-element of STRE (CCCCT) is crucial for the *GmCHX1* promoter in responding to salt stress. Therefore, *GmCHX1* promoter from PI 483460B (with STRE), Hutcheson (without STRE), and Hutcheson mutant (Hutcheson-mut, with STRE) were constructed into pCAMBIA3301 to drive the *β-glucuronidase* (*GUS*) gene in soybean hairy root. Soybean composite transgenic plants were developed according to Kereszt *et al.* (2007). The composite plants were treated with 1/4 Hoagland solution containing 0 or 100 mM NaCl for 24 h. GUS histochemical staining of hairy roots was conducted as described previously (Jefferson *et al.*, 1987). qRT-PCR of *GUS* gene was performed in hairy roots under the control and NaCl treatment conditions. The primer pairs used in the assay are listed in Supplementary Table S1.

### Statistical analysis

Phenotypic variations of LSS, CC_CK, CC_Salt, and CCR were analysed using the GLM procedure in SAS version 9.2 (SAS Institute, Inc., Cary, NC, USA). Broad-sense heritability was estimated as *H*^2^=σ^2^_g_/[σ^2^_g_+(σ^2^_ge_/*e*)+σ^2^/*re*], where σ^2^_g_ is the genetic variance, σ^2^_ge_ is the genotype by environment interaction variance, σ^2^ is the error variance, *r* is the number of replicates, and *e* is the number of environments. Duncan’s multiple range test or Student’s *t-*test was used to estimate the significance of difference and Pearson’s correlation (with SAS software) was used to estimate correlation coefficients.

## Results

### Identification of new soybean salt tolerance genetic resources

**Fig. 1.**
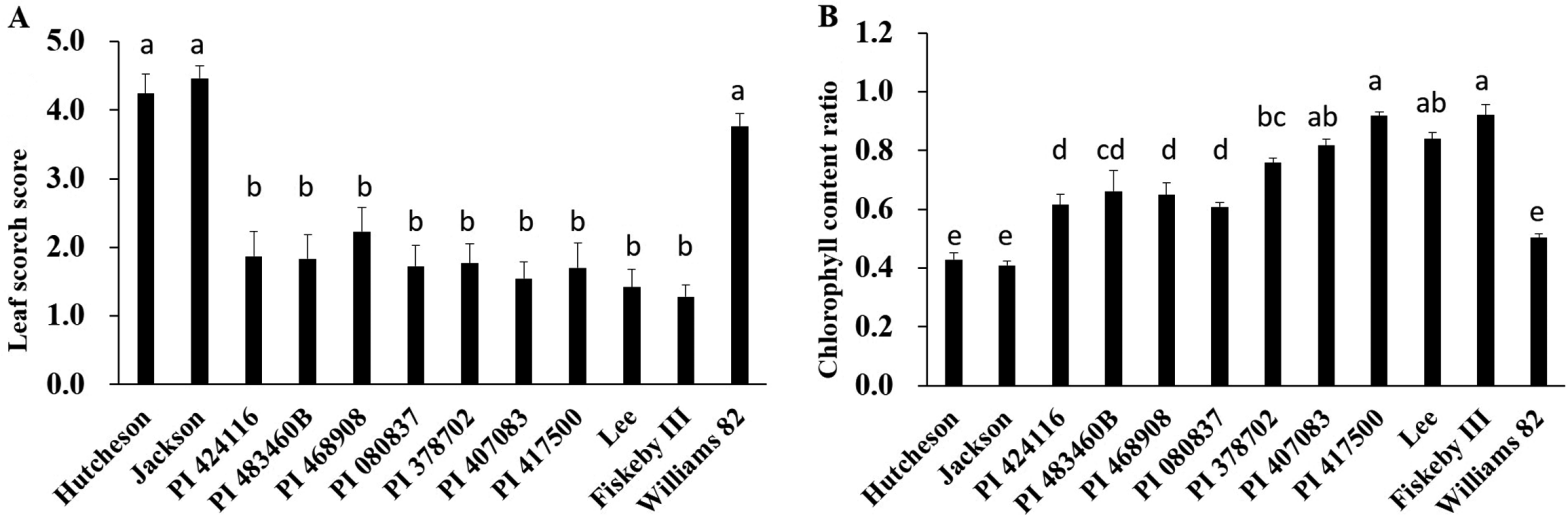
Leaf scorch score (LSS) and chlorophyll content ratio (CCR) of the selected salt-tolerant soybean lines under salt stress conditions. (A) LSS rated from 1 to 5, where 1 is no apparent chlorosis and 5 is dead. (B) CCR calculated as ratio of chlorophyll contents after and before salt treatment. The selected salt-tolerant soybean lines, salt-sensitive checks (Hutcheson, Jackson, and Williams 82), and salt-tolerant checks (Lee and Fiskeby III) were treated with 100 mM NaCl for 3 weeks before LSS and CCR evaluation. Data represent the mean ±SD of three biological replicates. Significant differences (a, b, c, d, or e above the bars) were identified by Duncan’s multiple range test at *P*=0.05.

**Fig. 2.**
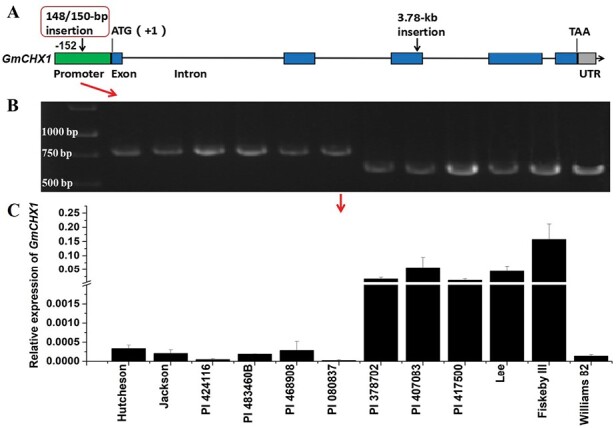
Characterization of the previously identified functional variation in *GmCHX1* promoter and the corresponding relative expression of *GmCHX1* in the selected salt-tolerant soybean lines and checks. (A) Graphical representation of the gene model and the previously identified functional variations of *GmCHX1*. (B) The genotype of the functional variation (148/150-bp insertion) in the *GmCHX1* promoter of the corresponding soybean lines in (C). (C) The relative expression of *GmCHX1* in the selected salt-tolerant soybean lines and checks under non-stress condition. Root samples were collected at vegetative growth stage V2 for qRT-PCR. The expression of *GmCHX1* is relative to the house-keeping gene *GmUKN1* (*Glyma.12G020500*). Data represent the mean ±SE of three replicates with three technical replicates for each sample.

In our previous study, seven soybean accessions were identified as new soybean salt-tolerant sources (Do *et al.*, 2019). Here, we confirmed the tolerant phenotype of the seven soybean lines showing significantly low LSS and high CCR compared with salt-sensitive checks under salt stress (Fig. 1A, B). We found all seven lines were carrying the salt-sensitive genotype of markers (Salt-20, Salt14056, and Salt11655) genotyped by KASP assays (Table 1) in *GmCHX1*. On further evaluation of the previously identified functional variations, the 148/150-bp InDel and 3.78-kb retrotransposon insertion (Fig. 2A) (Guan *et al.*, 2014; Qi *et al.*, 2014), four of the seven salt-tolerant lines (PI 424116, PI 483460B, PI 468908, PI 080837) were identified as having the 148/150-bp insertion (salt sensitive variation) in *GmCHX1* promoter, the same as the salt-sensitive checks (Hutcheson and Jackson) (Fig. 2B). The 3.78-kb retrotransposon insertion was not detected in the third exon of *GmCHX1* of the seven lines, and the insertion was only present in Williams 82 (Supplementary Fig. S1A, B).

**Table 1.** Phenotype and genotype of selected soybean salt-tolerant lines

PI number	Species	Phenotype for salt stress	Genotypes of GBMs^*a*^ in *GmCHX1*		
			Salt-20	Salt14056	Salt11655
PI 518664 (Hutcheson)	*Glycine max*	Sensitive check	WT	WT	WT
PI 548657 (Jackson)	*Glycine max*	Sensitive check	WT	WT	WT
PI 424116	*Glycine soja*	Tolerant	WT	WT	WT
PI 483460B	*Glycine soja*	Tolerant	WT	WT	WT
PI 468908	*Glycine max*	Tolerant	WT	WT	WT
PI 080837	*Glycine max*	Tolerant	WT	WT	WT
PI 378702	*Glycine soja*	Tolerant	WT	WT	WT
PI 407083	*Glycine soja*	Tolerant	WT	WT	WT
PI 417500	*Glycine max*	Tolerant	WT	WT	WT
PI 548656 (Lee)	*Glycine max*	Tolerant check	Mut	Mut	Mut
PI 438471 (Fiskeby III)	*Glycine max*	Tolerant check	Mut	Mut	Mut
PI 518671 (Williams 82)	*Glycine max*	Sensitive	WT	WT	WT

^*a*^ GBMs, gene-based markers.

The KASP assays of Salt-20, Salt14056, and Salt11655 were designed on SNPs in promotor, the third intron, and the fifth exon of *GmCHX1*, respectively.

The expression level of *GmCHX1* under non-stress conditions was examined. As expected, the expression level of *GmCHX1* was well-correlated with the presence/absence of the 148/150-bp insertion in the promoter under non-stress condition (Fig. 2B, C). The presence of the 148/150-bp insertion in the four lines PI 424116, PI 483460B, PI 468908, and PI 080837 led to significantly lower *GmCHX1* expression, compared with the lines without the 148/150-bp deletion, under non-stress condition (Fig. 2B, C). Altogether, these results suggest that these fours lines carry new salt tolerance gene(s) or new alleles of *GmCHX1*.

### Phenotypic variance and inheritance of salt tolerance traits in the recombinant inbred line population from Williams 82 × PI 483460B

**Fig. 3.**
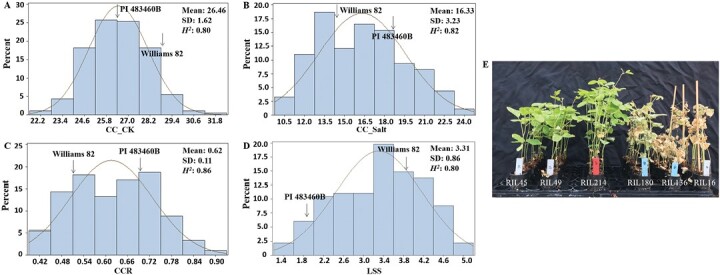
Phenotypic evaluation of salt tolerance representative traits in 182 RILs derived from a cross between Williams 82 and PI 483460B. (A) Phenotypic distribution of chlorophyll content before NaCl treatment (CC_CK). (B) Phenotypic distribution of chlorophyll content after 100 mM NaCl treatment (CC_Salt). (C) Phenotypic distribution of chlorophyll content ratio (CCR). (D) Phenotypic distribution of leaf scorch score (LSS) after 100 mM NaCl treatment. (E) Representative RILs with extreme phenotype for salt tolerance in leaf scorch.

To investigate if there was any new salt tolerance locus in these four newly identified lines, PI 483460B was selected for representative study. A RIL population derived from Williams 82 × PI 483460B was used to identify the above suggested new salt tolerance QTL/genes in soybean. The two parents, Williams 82 and PI 483460B, showed significant differences in salt tolerance as expected (Supplementary Fig. S2A). Significant variations in the four salt tolerance representative traits, including LSS, CCR, CC_CK, and CC_Salt, were observed in the RIL population with high heritability (0.80–0.86) (Supplementary Fig. S2B; Supplementary Table S2; Fig. 3A–D). The CC_CK value in Williams 82 was higher than in PI 483460B, and the opposite result for CC_Salt was observed after salt treatment (Fig. 3A, B). We observed transgressive segregations in these salt tolerance representative traits in this RIL population (Fig. 3C–E). A strong positive correlation between CC_Salt and CCR (*r*=0.95, *P*<0.001) and a relatively weak positive correlation between CC_Salt and CC_CK (*r*=0.39, *P*<0.001) were detected (Supplementary Table S3). No significant correlation was found between CC_CK and CCR. As expected, LSS had a significantly negative correlation (*r*≤-0.86, *P*<0.001) with CCR and CC_Salt (Supplementary Table S3).

### Quantitative trait locus mapping for salt tolerance in soybean recombinant inbred line population

**Fig. 4.**
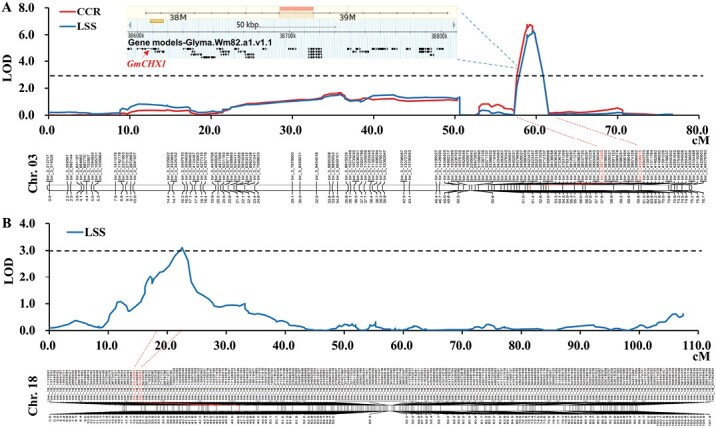
Graphical representation of QTL for salt tolerance mapped in the RIL population. (A) QTL *qSalt_Gm03* associated with salt tolerance on chromosome 3. The candidate genes for *qSalt_Gm03* are shown in the upper part of the figure and the red arrow indicates *GmCHX1*. (B) QTL *qSalt_Gm18* associated with salt tolerance on chromosome 18. CCR, chlorophyll content ratio; LSS, leaf scorch score. The significance threshold of the logarithm of the odds (LOD) values estimated by 1000 genome-wide permutations tests for CCR and LSS was <3.0.

QTL mapping was conducted to identify the genetic loci associated with CCR and LSS in the Williams 82 × PI 483460B population. A major locus, named as *qSalt_Gm03*, was mapped on Chr. 03 as being significantly associated with CCR and LSS, explaining 16.3% and 17.6% of the phenotypic variation (PVE, *R*^*2*^), respectively (Fig. 4A; Table 2). *qSalt_Gm03* is located at the same genomic region as the previously identified salt tolerance gene *GmCHX1* (Fig. 4A). A new locus on Chr. 18, denoted as *qSalt_Gm18*, was identified as being significantly associated with LSS, with LOD of 3.11 and *R*^*2*^ of 8.3% (Fig. 4B; Table 2). Both loci associated with salt tolerance had the donor alleles from salt-tolerant parent, PI 483460B (Table 2). No significant epistatic interaction was detected between *qSalt_Gm03* and *qSalt_Gm18*.

**Table 2.** QTL associated with salt tolerance-related traits was mapped in the RIL population of Williams 82 × PI 483460B

QTL	Traits^*a*^	Chr	LOD	Flanking markers^*b*^	Nearest marker	Position (cM)	Additive effect	*R* ^*2*^ (%)^*c*^	Donor^*d*^
*qSalt_Gm03*	CCR	3	6.97	bin_3_38712409 bin_3_40006018	bin_3_39385251	59.18	0.05	17.6	PI 483460B
	LSS	3	6.42	bin_3_38712409 bin_3_40006018	bin_3_39580451	59.51	-0.36	16.3	PI 483460B
*qSalt_Gm18*	LSS	18	3.11	bin_18_4215290 bin_18_5198448	bin_18_4745563	22.51	-0.26	8.3	PI 483460B

^*a*^ Traits for QTL mapping: CC_Salt: chlorophyll content after 100 mM NaCl treatment; CCR, chlorophyll content ratio where CCR=CC_Salt/CC_CK; LSS: leaf scorch score after 100 mM NaCl treatment.

^*b*^ The flanking makers delimiting the QTL regions based on the logarithm of odds (LOD) score distribution in Fig. 4.

^*c*^ The percentage contribution of the QTL to the total phenotypic variations for the respective traits.

^*d*^ The parent from which the favorable alleles of the QTL came.

### Identification of the salt-inducible expression of *GmCHX1* responsible for *qSalt_Gm03* in the four newly identified salt-tolerant lines including PI 483460B

**Fig. 5.**
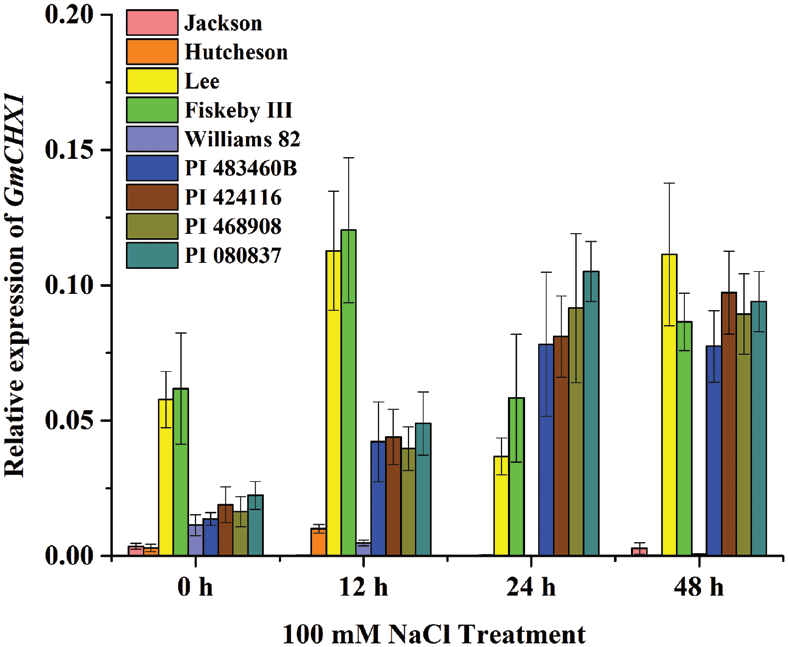
Relative gene expression level of *GmCHX1* in response to salt stress in the selected soybean lines. Relative expression of *GmCHX1* under 100 mM NaCl treatment for 0, 12, 24, and 48 h in the two parental lines (Williams 82 and PI 483460B), three new salt-tolerant lines (PI 424116, PI 468908, PI 080837), sensitive checks (Hutcheson and Jackson), and tolerant checks (Lee and Fiskeby III). Root samples were collected for qRT-PCR at vegetative growth stage V2 after 100 mM NaCl treatment. Data represent the mean ±SE of three replicates.

To determine whether *GmCHX1* underlies the *qSalt_Gm03* locus in PI 483460B and the other three lines, the expression levels of *GmCHX1* were examined in these four lines with five checks in response to salt treatment. As expected, under the control condition (0 h), the two tolerant checks (Lee and Fiskeby III) without the 148/150-bp insertion in the *GmCHX1* promoter showed significantly higher *GmCHX1* expression compared with the other four lines with the insertion (Fig. 5). In response to NaCl treatment from 12 to 48 h, Lee and Fiskeby III showed consistent high expression of *GmCHX1*, and the sensitive checks (Jackson and Hutcheson) showed consistent low expression of the gene (Fig. 5). Interestingly, the expression of *GmCHX1* in the four new salt-tolerant lines was induced after 12 h of NaCl treatment and reached comparable expression levels in Lee and Fiskeby III at 24 h and 48 h, respectively, after the treatment (Fig. 5). These results suggest that a new functional allele of *GmCHX1* is responsible for the *qSalt_Gm03* locus in these four new salt-tolerant lines.

**Fig. 6.**
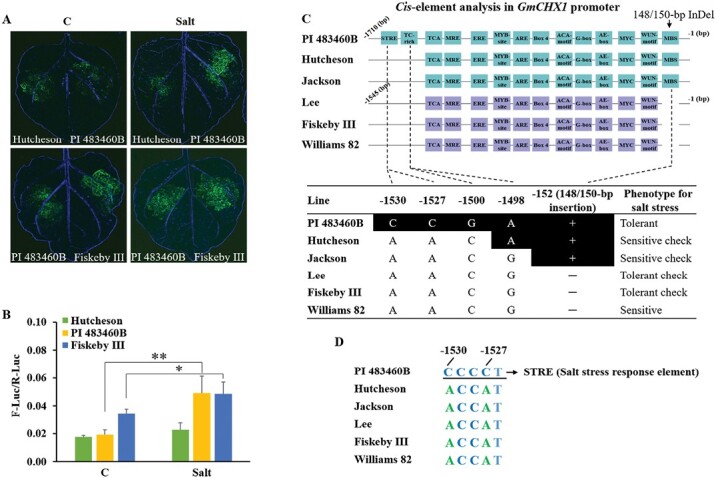
Different promoter activity and DNA sequence variations in *GmCHX1* promoter were detected in the selected soybean lines. (A) Representative images showing the luciferase activities of tobacco leaves infiltrated with an *Agrobacteria* strain harboring the corresponding reporter constructs with and without salt treatment. The *Luciferase* gene was driven by *GmCHX1* promoter (1.7 kb) cloned from Hutcheson, PI 483460B, and Fiskeby III. Tobacco plants were under control (C) or 100 mM NaCl (Salt) condition for 48 h after leaf infiltration of the transient expression constructs. (B) Quantitative luciferase activities of samples shown in (A). A dual-luciferase assay was used to quantify the luciferase activity, and the relative firefly luciferase (F-Luc) activity was normalized to *Renilla* luciferase (R-Luc) activity. Data represent the mean ±SD of three replicates. Significant difference was tested by Student’s *t-*test. (C) c*is*-element analysis of the *GmCHX1* promoters in the selected soybean lines. The physical positions of the nucleotides were numbered according to the reference genome (Williams 82) version Wm82.a2. +/− indicates the presence/absence of the insertion. (D) Two SNPs responsible for the presence/absence of a *cis*-element of STRE (CCCCT).

To understand the regulation of the salt-inducible expression of *GmCHX1* in these four new salt-tolerant lines, we cloned the promoters of *GmCHX1* from Hutcheson, PI 483460B (representing the four new salt-tolerant lines), and Fiskeby III into a luciferase reporter construct to evaluate the promoter activity in tobacco leaves using the dual-luciferase transcriptional activity assay. Under control condition, *GmCHX1* promoter of Fiskeby III had significant higher transcriptional activity compared with those of Hutcheson and PI 483460B (Fig. 6A, B). After treating the tobacco leaves transformed with promoter*::LUC* constructs for 48 h, we observed significant increased transcriptional activity of *GmCHX1* promoter in PI 483460B but not in Hutcheson and Fiskeby III (Fig. 6A, B). These results suggest that the salt-inducible expression pattern of *GmCHX1* in these four new salt-tolerant lines is due to new variations in the promoter region of the gene instead of variations in other genes that transcribe *GmCHX1*.

### Characterization of the DNA variations in the *GmCHX1* promoters

We initially sequenced and analysed the promoters of *GmCHX1* in six soybean lines, including Jackson, Hutcheson, PI 483460B, Lee, Fiskeby III, and Williams 82 (Supplementary Table S4). Among these six lines, abundant SNPs/InDels were found, in addition to the previously reported 148/150-bp InDel in *GmCHX1* promoter region (Supplementary Table S4). Twelve unique SNPs were identified in PI 483460B, which could be responsible to the salt-inducible expression of *GmCHX1* (Supplementary Table S4). *cis*-element analysis was performed to identify all the potential transcription factor/enhancer binding sites in the *GmCHX1* promoters of the six soybean lines (Fig. 6C; Supplementary Table S5). The *GmCHX1* promoter in PI 483460B was identified as having two unique *cis*-elements (TC-rich motif and STRE *cis*-element) due the unique SNPs in PI 483460B at positions of −1500(C/G)/−1498(G/A) and −1530(A/C)/−1527(A/C), respectively (Fig. 6C, D; Supplementary Table S5). The STRE motif has been previously reported to respond to salt stress and could be responsible for the salt-inducible expression of *GmCHX1* in PI 483460B (Schuller *et al.*, 1994; Martinez-Pastor *et al.*, 1996). Furthermore, to investigate if the other three new salt-tolerant lines share the same *cis*-element features at PI 483460B, we conducted DNA sequencing and sequence analysis. As expected, the STRE motif were also found in *GmCHX1* promoter in the other three new salt-tolerant lines due to the same variation as detected in PI 483460B (Supplementary Fig. S3A, B).

### Importance of STRE *cis*-element in the *GmCHX1* promoter in response to salt stress

**Fig. 7.**
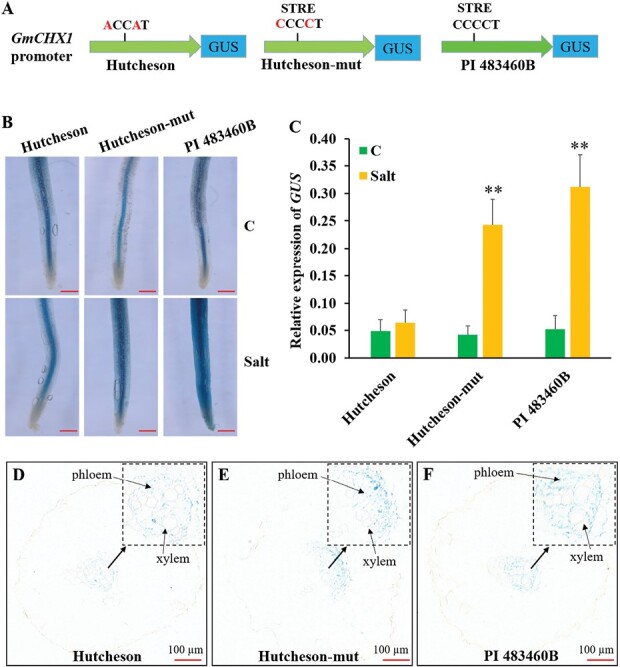
Effects of the two SNPs in the STRE *cis*-element in regulation of the salt-inducible *GmCHX1* expression. (A) Transgenic constructs to test the function of DNA variations in the STRE *cis*-element of the *GmCHX1* promoters. The Hutcheson type *GmCHX1* promoter was cloned directly from Hutcheson genomic DNA, and the Hutcheson-mut type *GmCHX1* promoter was generated by mutating the original Hutcheson promoter from ACCAT into CCCCT at the STRE site. The PI 483460B type *GmCHX1* promoter was cloned directly from PI 483460B genomic DNA. Promoters were cloned from a 1.7-kb region upstream the *GmCHX1* gene start codon (ATG). (B) GUS staining in soybean hairy roots transformed with the transgenic construct from (A) under control and salt stress conditions. Scale bar: 1 mm. (C) *GUS* gene expression in soybean hairy roots from (B). Data represent the mean ±SD of three replicates. Significance of difference was tested by Student’s *t-*test. (D–F) *GUS* expression and staining in specific tissue driven by Hutcheson type (D), Hutcheson-mut type (E), and PI 483460B type (F) *GmCHX1* promoter. Scale bar: 100 µm. Soybean composite plants with transgenic hairy roots were under control (C) or 100 mM NaCl (Salt) for 24 h.

To test whether the STRE *cis*-element is responsible to the salt-inducible expression of *GmCHX1* in the four new salt-tolerant lines, a promoter::*GUS* assay was performed in soybean transgenic hairy roots with the *GUS* gene driven by the *GmCHX1* promoter of Hutcheson type (ACCAT), Hutcheson-mut (CCCCT, STRE, Hutcheson promoter backbone), and PI 483460B type (CCCCT, STRE) (Fig. 7A). As expected, no expression induction of *GUS* treated by NaCl was observed in the hairy roots transformed with the Hutcheson promoter::*GUS* construct, while significant induction of *GUS* expression was identified in the hairy roots transformed with the Hutcheson-mut promoter::*GUS* or PI 483460B promoter::*GUS* construct (Fig. 7B, C). This result confirmed the hypothesis that variation in the STRE element (ACCAT to CCCCT) is responsible for the salt-inducible expression of *GmCHX1* under salt stress in the four new salt-tolerant lines.

Additionally, we observed GUS stain in the phloem and xylem cells of both control and salt-treated transgenic hairy roots transformed with all three promoter::*GUS* plasmids (Fig. 7D–F). This result suggests that the STRE motif in *GmCHX1* promoter does not affect the previously reported phloem and xylem tissue-specific expression of *GmCHX1* under salt stress (Guan *et al.*, 2014), and the tissue-specific expression of *GmCHX1* could be regulated by other common *cis*-elements shared with all alleles.

## Discussion

### New genetic resource for salt tolerance in soybean

Continuing efforts towards the identification of salt-tolerant germplasm and the cloning of salt-tolerant gene(s) is providing new genetic resources for salt tolerance improvement in crops, including soybean (Hamwieh *et al.*, 2011; Guan *et al.*, 2014; Qi *et al.*, 2014; Do *et al.*, 2016, 2018; Li *et al.*, 2017). In soybean, QTL/gene mapping work for salt tolerance was initiated using 106 RILs derived from ‘S-100’ (salt tolerant) × ‘Tokyo’ (salt sensitive) (Lee *et al.*, 2004). Several new soybean salt-tolerant germplasm lines were identified after large-scale salt tolerance evaluation, such as JWS156-1, W05, Tiefeng 8, Fiskeby III, IT162669, and NY36-87 (Luo *et al.*, 2005; Hamwieh and Xu, 2008; Chen *et al.*, 2013; Ha *et al.*, 2013; Qi *et al.*, 2014; Do *et al.*, 2018; Cho *et al.*, 2021; Guo *et al.*, 2021). In this study, seven soybean lines were confirmed as salt-tolerant germplasm (Fig. 1), and four of them (PI 424116, PI 483460B, PI 468908, and PI 080837) were identified as untapped resources for potential novel determinants of salt tolerance other than *GmCHX1* (Fig. 2; Table 1).

A major salt tolerance gene, *GmCHX1*, was previously mapped and cloned from soybean salt-tolerant germplasm (Guan *et al.*, 2014; Qi *et al.*, 2014; Do *et al.*, 2016). Additionally, some putative QTLs for salt tolerance with relatively minor effects were reported on Chrs 07, 08, 13, 15, 18, and 19 in soybean (Lee *et al.*, 2004; Chen *et al.*, 2008; Do *et al.*, 2018). In this study, a mapping population of 182 RILs derived from a cross of Williams 82 × PI 483460B was used to detect QTLs for salt tolerance (Fig. 3). A new salt tolerance locus, *qSalt_Gm18*, was mapped on Chr. 18 (Fig. 4B; Table 2), which was at least 7.5 Mb away from the salt tolerance-related QTL on Chr. 18 reported by Chen *et al.* (2008) and Do *et al.* (2019). The major salt tolerance locus in the newly identified four soybean lines is the same as *GmCHX1* with a novel salt-inducible promoter (Fig. 4A; Table 2) (Lee *et al.*, 2004; Guan *et al.*, 2014; Qi *et al.*, 2014; Do *et al.*, 2018). After screening more than 300 soybean diverse lines (representing the USDA soybean germplasm collection), no new major salt tolerance locus was identified (Do *et al*., 2019). These results suggest that there might be a few other major salt tolerance loci like *GmCHX1* in soybean germplasm and further improvement of salt tolerance in soybean may rely on gene pyramiding and gene-editing techniques.

### New allele of *GmCHX1* with a potential efficient energy cost for salt tolerance

Soybean germplasm is distributed widely in the world and exhibits genetic diversity for salt tolerance (Phang *et al.*, 2008; Wu *et al.*, 2014; Valliyodan *et al.*, 2016; Chen *et al.*, 2018). Molecular markers were developed to clone the major salt tolerance gene, *GmCHX1*, for marker-assisted selection in a soybean breeding program (Patil *et al.*, 2016). The current study showed the expression of *GmCHX1* was successively induced in PI 483460B and three other new salt-tolerant lines after 12 h of NaCl treatment, reaching a similar level to the salt-tolerant checks, Lee and Fiskeby III (Fig. 5). The dual-luciferase reporter assay confirmed the function of *GmCHX1* promoter in salt-inducible expression of the gene in PI 483460B (Fig. 6A, B). DNA sequence alignment and *cis*-element analysis discovered a unique STRE *cis*-element in the *GmCHX1* promoter of PI 483460B, which was confirmed to be responsible for the salt-inducible expression of *GmCHX1* in a subsequent promoter::*GUS* assay (Fig. 7A, B, C). This discovery is consistent with the STRE motif responding to salt stress as previously reported (Schuller *et al.*, 1994; Martinez-Pastor *et al.*, 1996). Natural variations in the promoter affecting its expression pattern were also detected in *GsERD15B* (*early responsive to dehydration 15B*) and *SlSOS1* (*Salt overly sensitive 1*) for salt tolerance (Jin *et al.*, 2021; Wang *et al*., 2021). The STRE motif does not change the tissue expression profile of *GmCHX1* promoter in phloem and xylem cells and the tissue-specific expression of *GmCHX1* could be regulated by other common *cis*-elements shared with all alleles (Fig. 7D–F).

Qi *et al*. (2014) revealed that elimination of the salt tolerance gene in salt-sensitive germplasm could be due to negative selection when its function was not required under an unstressed environment. The consistent high expression of the resistance/tolerance gene allele could have extra energy cost and metabolic burden and even toxic effects to the host plants. A trade-off can often occur between the benefits of using the consistent high-expression resistance/tolerance genes and a yield penalty (Gururani *et al.*, 2012; Vyska *et al.*, 2016). The *GmCHX1* promoter from PI 483460B with unique STRE motif identified in the current study can accurately control *GmCHX1* expression to reduce adverse effects of salt stress on soybean plants when needed. Thus, the novel *GmCHX1* allele with an accurate gene expression switch in the present study could provide an optimal strategy to improve salt tolerance and avoid a yield penalty under non-stress conditions.

## Supplementary data

The following supplementary data are available at *JXB* online.

Fig. S1. The genotype of 3.78-kb insertion in the third exon of *GmCHX1*.

Fig. S2. Phenotype of leaf scorch and leaf scorch ranking in soybean lines under salt stress.

Fig. S3. The sequence alignment of *GmCXH1* promoter for the other three new salt-tolerant soybean lines.

Table S1. The primer pairs were used in this study.

Table S2. ANOVA was performed for chlorophyll content and leaf scorch traits in the RIL population.

Table S3. Pearson correlation coefficients among chlorophyll content traits and leaf scorch score.

Table S4. The sequence polymorphism in *GmCHX1* promoter among the parental lines and checks.

Table S5. The information for stress-related *cis*-elements in *GmCXH1* promoter predicted by PlantCARE database.

erad404_suppl_Supplementary_Figure_S1-S3_Tables_S1-S3

erad404_suppl_Supplementary_Tables_S4-S5

## Data Availability

All data supporting the findings of this study are available within the paper and its supplementary data published online.
