# Supplementary material for: A novel natural variation in the promoter of GmCHX1 regulates conditional gene expression to improve salt tolerance in soybean
Source: J Exp Bot. 2023 Oct 21;75(3):1051–62. doi: 10.1093/jxb/erad404 (PMC10837011; doi:10.1093/jxb/erad404)
Supplement: erad404_suppl_Supplementary_Figure_S1-S3_Tables_S1-S3 [file erad404_suppl_supplementary_figure_s1-s3_tables_s1-s3.pdf]

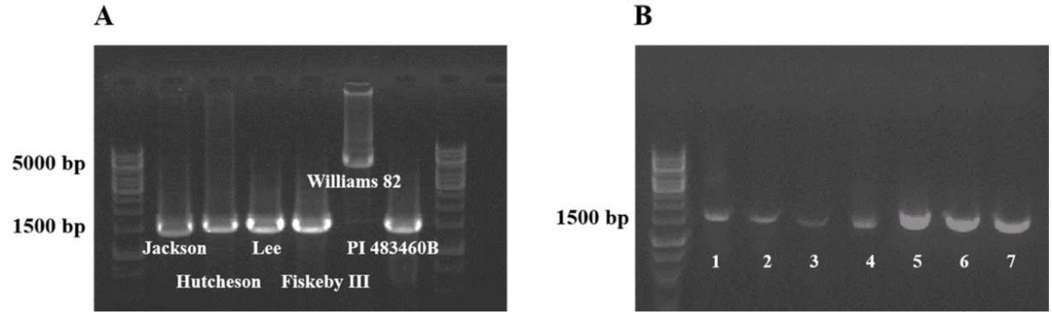

Supplementary Figure S1 The genotype of 3.78-kb insertion in the third exon of *GmCHX1*. **(A)** Detection of 3.78-kb insertion in *GmCHX1* among the checks and parental lines Williams 82 and PI 483460B. **(B)** Detection of 3.78-kb insertion in *GmCHX1* among the seven novel salt-tolerant lines. The number of 1-7 represents PI 424116, PI 483460B, PI 468908, PI 080837, PI 378702, PI 407083, and PI 417500, respectively.

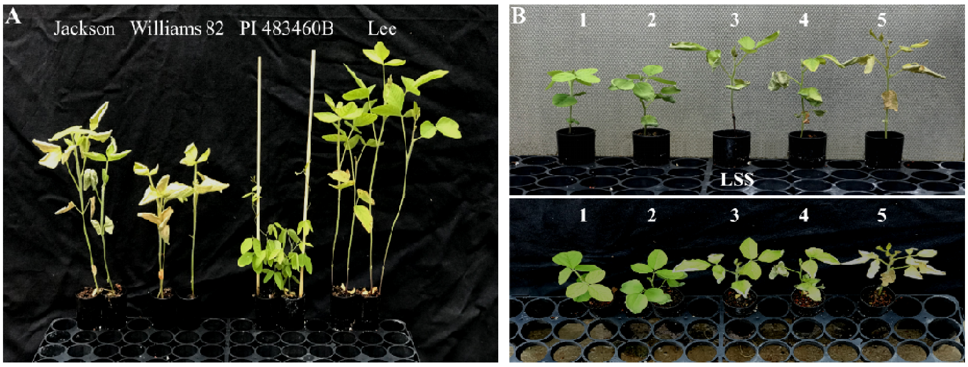

Supplementary Figure S2 Phenotype of leaf scorch and leaf scorch ranking in soybean lines under salt stress. (A) Leaf scorch in soybean plants of Jackson (sensitive check), Williams 82, PI 483460B, and Lee (tolerant check) after 100 mM NaCl treatment for three weeks. (**B)** Leaf scorch ranking from 1 to 5 in the RIL population, where 1 = no apparent chlorosis; 2 = slight (25% of the leaves showed chlorosis); 3 = moderate (50% of the leaves showed chlorosis and some necrosis); 4 = severe chlorosis (75% of the leaves showed chlorosis and severe necrosis); and 5 = dead (leaves showed severe necrosis and were withered).

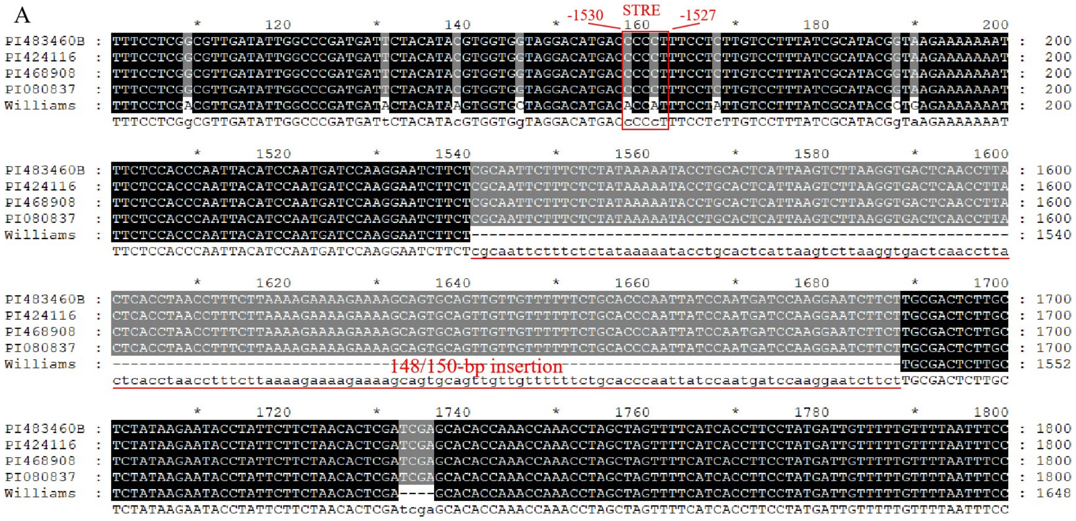

B

Name	Phenotype	-1676	-1668	-1638	-1579	-1557	-1549	-1542	-1530	-1527	-1520	-1500	-1498	-1457	-1429	-1409	-1398	-1385	-1334	-1310	-1303	-1287	-1276
PI483460B	Salt-tolerant	A	A	C	G	T	C	G	C	C	C	G	A	G	A	T	T	G	A	A	T	G	G
PI424116	Salt-tolerant	A	A	C	G	T	C	G	C	C	C	G	A	G	A	T	T	G	A	A	C	G	G
PI468908	Salt-tolerant	A	A	C	G	T	C	G	C	C	C	G	A	G	A	T	T	G	A	A	T	G	G
PI080837	Salt-tolerant	A	A	C	G	T	C	G	C	C	C	G	A	G	A	T	T	G	A	A	C	G	G
Williams 82	Salt-sensitive	T	T	A	A	A	A	C	A	A	A	C	G	C	G	G	A	A	T	T	T	T	T
**Name**	**Phenotype**	**-1270**	**-1132**	**-1103**	**-1101**	**-1100**	**-1078**	**-990**	**-971**	**-884**	**-871**	**-869**	**-868**	**-795**	**-641**	**-541**	**-446**	**-434**	**-415**	**-356**	**-290**	**-152 (148/150-bp insertion)**	**-103**
PI483460B	Salt-tolerant	T	A	T	T	T	G	G	C	C	A	A	A	T	C	A	A	A	G	G	T	+	TCGA
PI424116	Salt-tolerant	T	A	T	T	A	G	G	C	C	A	A	A	T	C	A	A	A	G	G	T	+	TCGA
PI468908	Salt-tolerant	T	A	T	T	A	G	G	T	C	A	A	A	T	C	A	G	A	G	G	T	+	TCGA
PI080837	Salt-tolerant	T	A	T	T	A	G	G	C	C	A	A	A	G	C	A	A	A	G	G	T	+	TCGA
Williams 82	Salt-sensitive	A	T	C	C	A	A	A	C	T	C	T	T	T	G	G	G	C	A	C	A	–	----

Supplementary Figure S3 The sequence alignment and variation detection in *GmCXH1* promoter for the other three new salt-tolerant soybean lines. (**A**) The two major sequence variations in *GmCXH1* promoter were detected in three new salt-tolerant soybean lines (PI 424116, PI 468908, PI 080837). Parental lines of PI 483460B and Williams 82 act as checks. STR (CCCCT) motif, responds to salt stress. (**B)** The sequence polymorphism in *GmCHX1 promoter* among the parental lines and three new salt-tolerant lines.

Supplementary Table S1 The primer pairs were used in this study

Primer Name	Sequence	Purpose
Pro-insertion-F	GGGTTGTGCCTAAATAGCA	Detect the 148/150-bp insertion
Pro-insertion-R	AAGGAAGAGCGTGGTTCA	Detect the 148/150-bp insertion
Exon-insertion-F	GCGGGAGTAATGTTATCGG	Detect the 3.78-kb insertion
Exon-insertion-R	AAGGTGGTGAAGAGAGCCA	Detect the 3.78-kb insertion
qPCR-GmCHX1-F	ACCTGGCAACATACCCTCA	Detect the expression of *GmCHX1*
qPCR-GmCHX1-R	TGGTCCACTCTTCATTCGG	Detect the expression of *GmCHX1*
GmUKN1-F	TGGTGCTGCCGCTATTTACTG	House-keeping gene for qPCR
GmUKN1-R	GGTGGAAGGAACTGCTAACAATC	House-keeping gene for qPCR
Pro-Luc-F	ctatagggcgaattgggtaccGAACAACTGGGTTAAGGCGTAAA	Dual-Luciferase reporter assay
Pro-Luc-R	atcgataccgtcgacctcgagGGCCAAAGACTCAGTGCTTGTT	Dual-Luciferase reporter assay
Pro-mut-GUS-F	TGCTAGGACATGACCCCCTTTC	Promoter-*GUS* in hairy root
Pro-mut-GUS-R	GAAAGGGGGTCATGTCCTAGCA	Promoter-*GUS* in hairy root
Pro-GUS-F	tatgaccatgattacgaattcGAACAACTGGGTTAAGGCGTAAA	Promoter-*GUS* in hairy root
Pro-GUS-R	ttaccctcagatctaccatggGGCCAAAGACTCAGTGCTTGTT	Promoter-*GUS* in hairy root
qPCR-GUS-F	CACGCCGTATGTTATTGCCG	Detect the expression of GUS gene
qPCR-GUS-R	CGGTGATATCGTCCACCCAG	Detect the expression of GUS gene

Supplementary Table S2 ANOVA were conducted for chlorophyll content and leaf scorch traits in the RIL population

Traits	Source	DF	Sum of Squares	Mean Square	F Value	*Pr* > F
CC_CK[a]	Replication	1	2483.58	2483.58	1127.13	<0.0001
	RILs	181	804.06	4.44	2.02	<0.0001
	Error	176	387.81	2.20		
	Corrected total	358	3741.74			
CC_Salt[b]	Replication	1	1663.52	1663.52	186.39	<0.0001
	RILs	181	3642.34	20.12	2.25	<0.0001
	Error	176	1570.78	8.92		
	Corrected total	358	6933.41			
CCR[c]	Replication	1	0.1	0.14	10.95	0.0011
	RILs	181	4.44	0.02	1.91	<0.0001
	Error	176	2.26	0.01		
	Corrected total	358	6.84			
LSS[d]	Replication	1	3.73	3.73	8.14	0.0048
	RILs	181	262.45	1.45	3.16	<0.0001
	Error	176	80.65	0.46		
	Corrected total	358	347.27			

[a]CC_CK: Chlorophyll content before 100 mM NaCl treatment.

[b]CC_Salt: Chlorophyll content after 100 mM NaCl treatment.

[c]CCR: Chlorophyll content ratio. CCR = CC_Salt/CC_CK.

[d]LSS: Leaf scorch score after 100 mM NaCl treatment.

Supplementary Table S3 Pearson correlation coefficients among chlorophyll content traits and leaf scorch score (LSS)

	CC_CK	CC_Salt	CCR	LSS
CC_CK	1.00			
CC_Salt	0.39***	1.00		
CCR	0.09^{ns}	0.95***	1.00	
LSS	-0.29***	-0.89***	-0.86***	1.00

Pearson correlation coefficients were calculated between different traits with significance levels of $P < 0.05$ (*), $P < 0.01$ (**), and $P < 0.001$ (***). The ns represents no significance. CC_CK: Chlorophyll content before 100 mM NaCl treatment. CC_Salt: Chlorophyll content after 100 mM NaCl treatment. CCR: Chlorophyll content ratio. CCR = CC_Salt/CC_CK. LSS: Leaf scorch score after 100 mM NaCl treatment.
